# Efficacy and safety of bemcentinib in patients with advanced myelodysplastic neoplasms or acute myeloid leukemia failing hypomethylating agents- the EMSCO phase II BERGAMO trial

**DOI:** 10.1038/s41375-023-02029-1

**Published:** 2023-09-21

**Authors:** A. S. Kubasch, P. Peterlin, T. Cluzeau, K. S. Götze, K. Sockel, R. Teipel, M. Jentzsch, H. Attalah, M. Sebert, F. Chermat, S. Gloaguen, M. Puttrich, M. Cross, M. Schneider, S. Kayser, D. Schipp, A. Giagounidis, I. Tirado-Gonzalez, A. Descot, A. van de Loosdrecht, A. Weigert, K. H. Metzeler, P. Fenaux, H. Medyouf, U. Platzbecker, L. Ades

**Affiliations:** 1 Department of Hematology, Hemostaseology, Cellular Therapy and Infectious Diseases, Leipzig University Hospital, Leipzig, Germany; 2 German MDS Study Group (D-MDS), Leipzig, Germany; 3 The European Myelodysplastic Neoplasms Cooperative Group (EMSCO), Leipzig, Germany; 4 Service d’hématologie Clinique, CHU de Nantes, Nantes, France; 5 CHU de Nice, Département d’Hématologie Clinique, Nice, France; 6 Department of Medicine III, Klinikum rechts der Isar, TUM, Munich, Germany; 7 Department of Internal Medicine I, University Hospital Carl Gustav Carus Dresden, TU Dresden, Dresden, Germany; 8 Groupe Francophone des Myélodysplasies, Paris, France; 9 Service d’Hématologie Seniors, Hopital Saint Louis, Assistance Publique-Hôpitaux de Paris and Paris Cité University, Paris, France; 10 GWT-TUD GmbH, Dresden, Germany; 11 Institute of Transfusion Medicine and Immunology, Medical Faculty Mannheim, Heidelberg University, German Red Cross Blood Service Baden-Württemberg-Hessen, Mannheim, Germany; 12 NCT Trial Center, National Center of Tumor Diseases, German Cancer Research Center (DKFZ), Heidelberg, Germany; 13 DS-Statistics, Rosenthal-Bielatal, Germany; 14 Department for Oncology, Hematology and Palliative Care, Marien Hospital Düsseldorf, Düsseldorf, Germany; 15 Georg-Speyer-Haus, Institute for Tumor Biology and Experimental Therapy, Frankfurt, Germany; 16 Amsterdam UMC, VU University Medical Center, Amsterdam, Cancer Center Amsterdam, Amsterdam, Netherlands; 17 Frankfurt Cancer Institute, Frankfurt am Main, Germany; 18 German Cancer Research Consortium, Frankfurt/Mainz partner site, DKFZ Heidelberg, Heidelberg, Germany

**Keywords:** Myelodysplastic syndrome, Myelodysplastic syndrome

## To the Editor:

Hypomethylating agents (HMAs) are the standard of care for patients with higher-risk myelodysplastic neoplasms (HR-MDS) and, in combination with venetoclax, for patients with acute myeloid leukemia (AML) who are not eligible for intensive chemotherapy (IC) or allogeneic stem cell transplantation (alloSCT). Nevertheless, responses occur only in around 50% of patients and are generally short-lived [1]. Thus, the majority of patients either do not respond to these agents or experience relapse, which associates a dismal outcome with a median survival of around 5 months [2]. Outside clinical trials, there are currently limited approved treatment options available for this patient population.

The receptor tyrosine kinase AXL is linked to the pathogenesis of myeloid malignancies and mediates chemotherapy resistance [3, 4]. Moreover, recent studies have shown that AXL inhibition enhances anti-leukemic immune responses [4, 5]. Given that AXL is known to be upregulated on leukemic MDS and AML stem cells [5–7]AXL inhibition has been explored as a potential new targeted therapy for patients with myeloid malignancies in several clinical trials [4, 5, 8].

The BERGAMO multicenter phase 2 trial (NCT03824080) evaluated the safety and efficacy of the oral, selective, small molecule AXL inhibitor bemcentinib (BEM) in patients with HR-MDS or AML not eligible for IC or alloSCT, refractory or relapsing after at least six cycles of azacitidine (AZA) or four cycles of decitabine (DAC). Patients were eligible if baseline bone marrow blast count by central morphology was ≥5% and ≥1 cytopenia according to IPSS-R was present. Patients received an initial loading dose of 400 mg BEM orally once daily administered on days 1–3 of cycle 1 and a maintenance dose of 200 mg BEM on days 4–28 of cycle 1 and in subsequent 28-day treatment cycles.

The primary efficacy endpoint was the overall hematological response rate (OHR) defined as complete response (CR), marrow complete response (mCR), partial response (PR), stable disease (SD) or hematologic improvement (HI) according to the modified IWG 2006 criteria [9] and 2017 European LeukemiaNet (ELN) recommendations, respectively [10], as assessed at week 17 after four BEM treatment cycles. In the intention-to-treat analysis, the primary hypothesis (OHR ≤ 5% vs. OHR > 5%) was tested by one sample binomial test. Time to event endpoints were analyzed by Kaplan–Meier method.

All patients who achieved CR, mCR, PR, SD or HI (HI-E, HI-P, HI-N) after the first four BEM treatment cycles were considered as responders and allowed to continue treatment for up to nine treatment cycles. Non-responding patients stopped BEM treatment after the first four cycles. Secondary endpoints were rate and grade of toxicity as measured by NCI CTCAE 5.0, overall survival (OS), progression-free-survival (PFS), time to treatment failure, duration of response (DOR) and best overall response. Exploratory analyses evaluated the role of potential molecular biomarkers to predict response to BEM treatment in MDS and AML. Responders and non-responders were compared with respect to presence of specific mutations by Fisher’s exact test.

From 2018 to 2020, a total of 57 patients (MDS = 26, AML = 31) were screened at ten different trial sites in Germany and France within the ‘European Myelodysplastic Neoplasms Cooperative Group‘ (EMSCO). Forty-five patients (MDS = 18, AML = 27) were included (Table 1A) and received at least one cycle of BEM and were eligible for safety and efficacy analyses. Patients’ median age was 79 years (range 62–86 years) and median baseline hemoglobin in the total cohort was 8.7 g/dl (range 6.6–12.5 g/dl) with 44% of MDS patients being red blood cells (RBC) transfusion-dependent compared to 52% of AML patients. Platelet transfusions dependency at baseline was observed in 33% of MDS and 41% of AML patients, respectively. Median bone marrow blast count at screening in the MDS and AML cohorts was 13% and 33%, respectively. MDS patients were classified as IPSS-R intermediate (11%), high (44%) and very high risk (33%) (*n* = 2, 11% missing). AML patients were classified as favorable (26%), intermediate (26%) and adverse risk (37%) (*n* = 3, 11% missing) according to ELN 2017 [10] prognostic system. Fourty-three patients (96%) had received prior therapy with AZA and two patients (4%) with DAC, of whom one patient (2%) was considered as HMA intolerant, 10 patients (22%) were refractory and 34 patients (76%) relapsed after initial response to HMA treatment. The median number of prior AZA or DAC cycles was 13 (range 5–50 cycles). Median time interval between HMA failure and start of BEM treatment was 64 days (range 2–943 days).

**Table 1:** **A**. Patient baseline characteristics and response rates in the BERGAMO trial. **B**. Molecular genetics at screening.

A			
Characteristic	Total (*n* = 45); number (%) or median [range]	HR-MDS patients (*n* = 18); number (%) or median [range]	AML patients (*n* = 27); number (%) or median [range]
Age	79 [62–86]	76 [62–84]	81 [72–86]
Gender			
*Female*	17 [38]	7 [39]	10 [37]
*Male*	28 [62]	11 [61]	17 [63]
MDS subtype			
*MDS-EB1*		2 [11]	
*MDS-EB2*		13 [72]	
*Missing*		3 [17]	
IPSS-R			
*Intermediate*		2 [11]	
*High*		8 [44]	
*Very high*		6 [33]	
*Missing*		2 [11]	
ELN risk category			
*Favorable*			7 [26]
*Intermediate*			7 [26]
*Adverse*			10 [37]
*Missing*			3 [11]
Hemoglobin (g/dl)	8.7 [6.6–12.5]	8.7 [7.2–11.0]	8.8 [6.6–12.5]
RBC transfusion dependency	22 [49]	8 [44]	14 [52]
Platelet count	25 [7–218]	34 [14–195]	22 [7–218]
Platelet transfusion dependency	17 [38]	6 [33]	11 [41]
Absolut neutrophil count	0.6 [0.02–16.11]	0.8 [0.10–6.42]	0.4 [0.02–16.11]
ORR at pEP	11 [24]	8 [44]	3 [11]
*CR*	1 [2]	1 [6]	0 (0)
*mCR*	5 [11]	5 [28]	0 (0)
*PR*	1 [2]	1 [6]	0 (0)
*SD*	4 [9]	1 [6]	3 [11]
One year OS (%)	28	54	n/a
One year PFS (%)	7	6	8
Median cycles given	4 [3–21]	6 [3–19]	4 [3–21]

B												
Type of somatic mutation (Subpopulation)		Frequency										Comparison of responders vs. non-responders (Fisher’s exact test)
		Total (*N* = 34)		MDS (*N* = 14)		AML (*N* = 20)		Non-responder (*N* = 24)		Responder (*N* = 10)		
	Mutation	*N*	%	*N*	%	*N*	%	*N*	%	*N*	%	
Screening	≥1 mutation	32	94.1	12	85.7	20	100	22	91.7	10	100	
	≥2 mutations	30	88.2	12	85.7	18	90.0	20	83.3	10	100	
	≥3 mutations	29	85.3	12	85.7	17	85.0	20	83.3	9	90.0	
	≥4 mutations	23	67.6	10	71.4	13	65.0	16	66.7	7	70.0	
	No mutations	2	5.9	2	14.3	0	0.0	2	8.3	0	0.0	
	ASXL1	17	50.0	8	57.1	9	45.0	12	50.0	5	50.0	1.000
	BCOR	2	5.9	1	7.1	1	5.0	1	4.2	1	10.0	0.508
	CBL	3	8.8	3	21.4	0	0.0	2	8.3	1	10.0	1.000
	CEBPA	4	11.8	1	7.1	3	15.0	3	12.5	1	10.0	1.000
	CSF3R	1	2.9	1	7.1	0	0.0	0	0.0	1	10.0	0.294
	CSNK1A1	1	2.9	0	0.0	1	5.0	0	0.0	1	10.0	0.294
	DDX41	3	8.8	1	7.1	2	10.0	2	8.3	1	10.0	1.000
	DNMT3A	8	23.5	3	21.4	5	25.0	4	16.7	4	40.0	0.195
	ETNK1	1	2.9	1	7.1	0	0.0	1	4.2	0	0.0	1.000
	ETV6	2	5.9	1	7.1	1	5.0	1	4.2	1	10.0	0.508
	EZH2	4	11.8	2	14.3	2	10.0	2	8.3	2	20.0	0.564
	FLT3	4	11.8	0	0.0	4	20.0	4	16.7	0	0.0	0.296
	GATA2	2	5.9	1	7.1	1	5.0	1	4.2	1	10.0	0.508
	GNB1	1	2.9	0	0.0	1	5.0	1	4.2	0	0.0	1.000
	IDH1	3	8.8	0	0.0	3	15.0	2	8.3	1	10.0	1.000
	IDH2	4	11.8	0	0.0	4	20.0	2	8.3	2	20.0	0.564
	JAK2	1	2.9	0	0.0	1	5.0	1	4.2	0	0.0	1.000
	JAK3	1	2.9	0	0.0	1	5.0	1	4.2	0	0.0	1.000
	KIT	1	2.9	0	0.0	1	5.0	1	4.2	0	0.0	1.000
	KRAS	4	11.8	2	14.3	2	10.0	3	12.5	1	10.0	1.000
	MPL	1	2.9	0	0.0	1	5.0	0	0.0	1	10.0	0.294
	NF1	1	2.9	1	7.1	0	0.0	1	4.2	0	0.0	1.000
	NPM1	4	11.8	0	0.0	4	20.0	4	16.7	0	0.0	0.296
	NRAS	6	17.6	3	21.4	3	15.0	4	16.7	2	20.0	1.000
	PHF6	3	8.8	1	7.1	2	10.0	2	8.3	1	10.0	1.000
	PRPF8	3	8.8	1	7.1	2	10.0	2	8.3	1	10.0	1.000
	PTPN11	1	2.9	0	0.0	1	5.0	1	4.2	0	0.0	1.000
	RAD21	1	2.9	1	7.1	0	0.0	0	0.0	1	10.0	0.294
	RUNX1	13	38.2	6	42.9	7	35.0	9	37.5	4	40.0	1.000
	SETBP1	3	8.8	3	21.4	0	0.0	2	8.3	1	10.0	1.000
	SF3B1	4	11.8	2	14.3	2	10.0	3	12.5	1	10.0	1.000
	SH2B3	1	2.9	1	7.1	0	0.0	0	0.0	1	10.0	0.294
	SRSF2	12	35.3	6	42.9	6	30.0	8	33.3	4	40.0	0.714
	**STAG2**	**6**	**17.6**	**3**	**21.4**	**3**	**15.0**	**2**	**8.3**	**4**	**40.0**	**0.048**
	TET2	10	29.4	4	28.6	6	30.0	8	33.3	2	20.0	0.683
	TP53	6	17.6	1	7.1	5	25.0	4	16.7	2	20.0	1.000
	U2AF1	3	8.8	1	7.1	2	10.0	3	12.5	0	0.0	0.539
	WT1	2	5.9	1	7.1	1	5.0	2	8.3	0	0.0	1.000
	ZRSR2	1	2.9	0	0.0	1	5.0	0	0.0	1	10.0	0.294

STAG2 mutation in bold is the only significant mutation.

Only 16 (MDS = 11, AML = 5) patients completed the first four BEM treatment cycles, reasons for premature study termination within this period were disease-related death (*n* = 6), investigator decision due to disease progression (*n* = 19) or occurrence of adverse events (*n* = 3), and withdrawal of consent (*n* = 1).

**Fig. 1:**
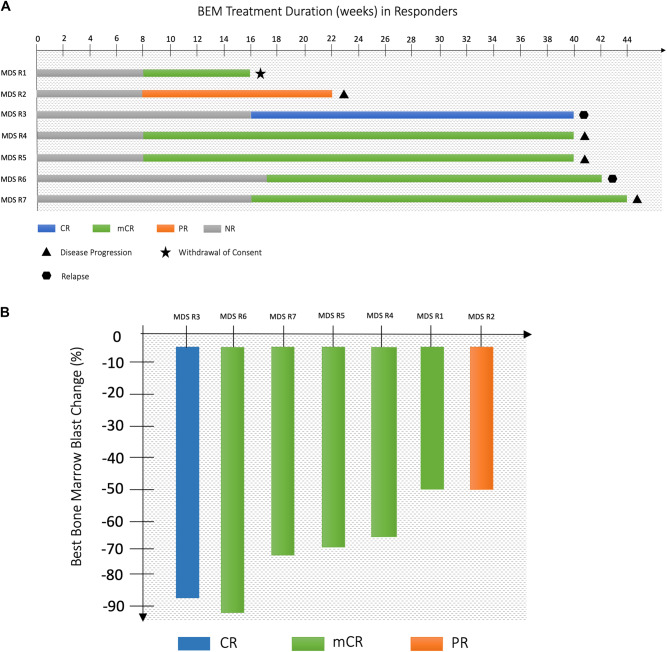
Treatment overview and response status. **A** Treatment overview in responders. Treatment duration and response status in MDS patients achieving a response. **B** Bone marrow blast change in responders. Best change from baseline in bone marrow blasts (in %) in MDS patients achieving a response.

The primary endpoint was met with 11/45 patients (24%) responding. Within the MDS cohort, a higher fraction of patients (8 out of 18 patients = 44%) achieved a response including 1 CR (6%), 5 mCR (28%), 1 PR (6%) and 1 SD (6%). In contrast, the AML cohort showed limited response to BEM, with only 3/27 patients (11%) exhibiting SD. During the entire treatment period, the median number of BEM treatment cycles in the total cohort was four (range, 3–21 cycles) and the median duration of BEM treatment was 10 weeks (range 1–95 weeks) (Fig. 1A). Four of the five MDS patients achieving mCR had a normal karyotype (80%) and 1 (20%) patient had a loss of the Y chromosome. Among the MDS non-responders (*n* = 10), 4/10 patients (40%) had a complex karyotype.

Patient disposition is provided in Table 1A. Molecular genetics (Table 1B) at study entry are in line with a poor-risk study population with advanced disease. The one-year OS rate in the whole cohort and HR-MDS subgroup was 28% and 54% respectively. The one-year PFS was 8% in AML patients compared to 6% in MDS patients. According to the log rank test, the PFS curves are significantly different between AML (median time to event (TTE) 8 weeks) and MDS (median TTE 22 weeks) subgroups (*p* = 0.012).

The median DOR to BEM was 25 and 12 weeks in MDS and AML patients, respectively (Fig. 1A). The best overall response for the complete study period in MDS patients was 56% (*n* = 10) including 1 CR, 5 mCR, 1 PR and 3SD (Fig. 1B). In the AML cohort, the OHR was 15% (*n* = 4) including 1 PR and 3SD.

Treatment with BEM was generally well tolerated. The grade 4 treatment-related adverse events (TRAEs) were neutrophil count decrease (*n* = 2), C-reactive protein increase (*n* = 1) and grade 3 TRAEs were ECG QT prolongation (*n* = 3) and one case each of anemia, thrombocytopenia, diarrhea, asthenia, LDH increase and decreased appetite. In the three cases of ECG QT prolongation, no action was taken (*n* = 2) or the drug was stopped (*n* = 1) with normalization of the ECG after treatment discontinuation.

At the time of data cut off, BEM-related G3-5 serious adverse events (SAEs) were reported in 14 out of 45 patients (31%). We saw grade 5 potentially BEM-related SAEs including acute kidney injury (*n* = 1) and disease progression (*n* = 2); no grade 4 events but 14 grade 3 events were reported in 12 patients including sepsis (2 events, 1 patient) and one patient each with pneumonia, acute kidney injury, periodontitis, febrile neutropenia, upper gastrointestinal hemorrhage, pneumonitis, abdominal pain, nausea, febrile bone marrow aplasia, bone pain, general physical health deterioration and headache. Twenty-five patients (56%) died during the study with the most common cause reported as disease progression (*n* = 18) not related to BEM.

To identify potential molecular patterns correlating with response to BEM, patients were centrally screened at baseline for somatic variants in 68 candidate genes associated with myeloid malignancies using a targeted next-generation sequencing gene panel (Table 1B). In exploratory analyses, mutations in *STAG2* [11], were significantly more frequent in responders (40%) compared to non-responders (8%) (*p* = 0.048, unadjusted Fisher’s exact test). STAG2 is part of the cohesion complex, frequently mutated in HR-MDS/sAML [12], that coordinates sister chromatid separation during cell division. Consequently, mutations in cohesion complex are linked to increased DNA damage repair defects [13], with STAG2 deficiency specifically associated with inducing interferon response via cGAS-STING pathway [14]. It is therefore tempting to speculate that *STAG2* mutations may correlate with improved response to BEM, by potentiating the immune-sensitizing effects of BEM, recently reported by our group in pre-clinical models of AML [5] or by mediating synthetic letality [15]. Further studies are required to specifically define the potential significance of *STAG2* mutation in the response to BEM.

In conclusion, this prospective study showed that BEM exhibits a good tolerability profile in a highly vulnerable patient population. In terms of efficacy, BEM displays moderate single-agent activity in this population, with early terminations due to disease progression. Nonetheless, despite the advanced disease stage and the small number of patients, responders were primarily seen in the MDS cohort, and the individuals with *STAG2* mutation prompting the need for further studies with BEM in selected cohorts of patients. Such a follow-up study will aim to better define which subgroup of MDS patients will benefit most from single agent or combinational BEM treatment and which role molecular biomarkers such as *STAG2* play in patient stratification.
